# Slow Mohs Micrographic Surgery and Reconstruction With Artificial Dermis for Atrophic Dermatofibrosarcoma Protuberans: A Case Report

**DOI:** 10.7759/cureus.64433

**Published:** 2024-07-12

**Authors:** Peiyun Ho, Meng-Han Shen

**Affiliations:** 1 Department of Dermatology, Wan Fang Hospital, Taipei Medical University, Taipei, TWN

**Keywords:** atrophic dfsp, dfsp, artificial dermis, slow-mohs micrographic surgery, atrophic dermatofibrosarcoma protuberans

## Abstract

This case report describes the utility of artificial dermis in reconstruction for atrophic dermatofibrosarcoma protuberans (DFSP) after slow Mohs micrographic surgery (MMS). A 34-year-old man presented as a slowly growing nodule from an *atrophic scar* on his right chest for over 10 years. The pathology report confirmed the diagnosis of atrophic DFSP. Further magnetic resonance imaging (MRI) revealed a 9.3 cm x 6.5 cm cutaneous-subcutaneous lesion with close contact with the pectoralis major muscle. The patient underwent a slow MMS, and we utilized a rotational flap in combination with synthetic xenogeneic artificial dermis to reconstruct the final 13 cm x 12 cm defect.

## Introduction

Dermatofibrosarcoma protuberans (DFSP) is a locally invasive malignant tumor characterized by infiltrative growth [1]. It commonly infiltrates underlying tissues such as subcutis, fascia, muscle, and periosteum, exhibiting unpredictable subclinical extension and local destructive capacity [2]. Variants of DFSP include myxoid, pigmented, granular cell, sclerosing, atrophic, and fibrosarcomatous types [3]. Atrophic DFSP can be mistaken for an atrophic scar or scleroderma due to its diverse clinical presentations and insidious progression, leading to delayed diagnosis in patients averaging three to five years [4]. The gold standard treatment for localized DFSP is complete surgical resection with microscopically negative margins [5]. Here, we present a case of atrophic DFSP involving the deep fascia, detected using slow Mohs micrographic surgery (MMS), followed by reconstruction with artificial dermis.

## Case presentation

**Figure 1:**
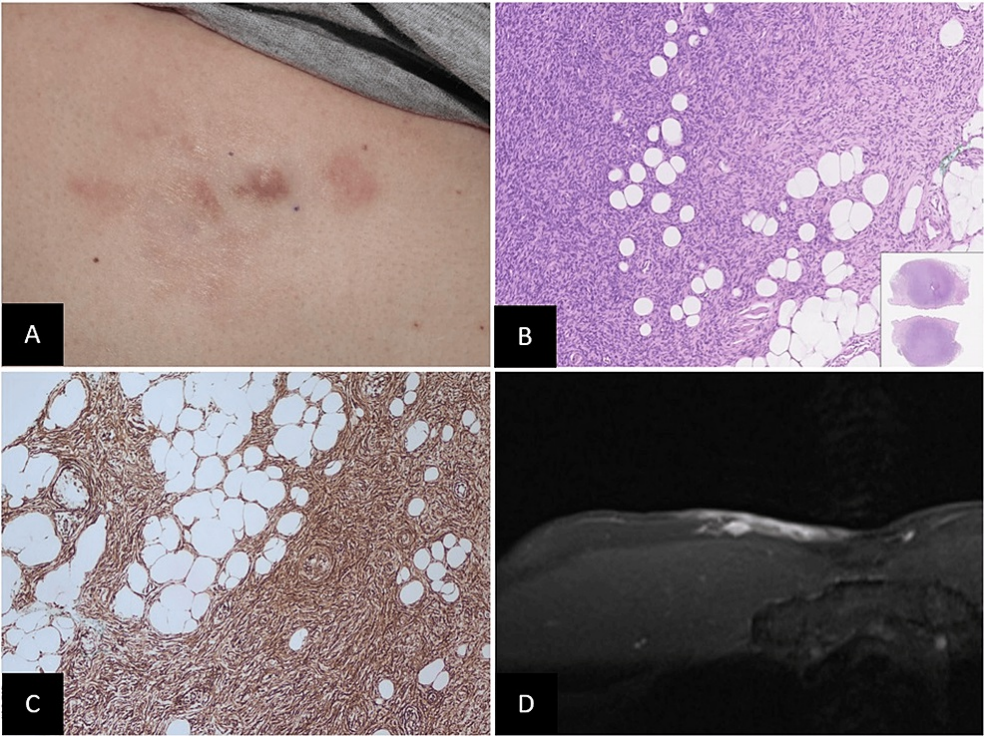
(A) An infiltrative indurated skin-colored to pinkish atrophic plaque with palpable elastic nodule on the right chest; (B) histologically showed a storiform pattern of uniform spindle cells diffusely infiltrating the dermis and the subcutis in a honeycomb pattern; (C) immunohistochemical stains demonstrated diffuse positivity for CD34 antigen in the spindle cells; (D) magnetic resonance imaging (MRI) demonstrated a 6.5 cm x 9.3 cm in maximum size and 1.0 cm in depth lesion with close contact to pectoralis major muscle.

A 34-year-old man presented to our dermatology clinic with a slowly growing nodule emerging from an *atrophic scar* on his right chest, which had developed over the past 10 years. The lesion appeared gradually during his twenties without any history of trauma or associated symptoms. On examination, we observed an 8 cm x 5 cm infiltrative, firm, skin-colored to pinkish plaque with a palpable elastic nodule (Figure 1A). An incisional biopsy revealed a storiform pattern of uniform spindle cells infiltrating the dermis and subcutis in a honeycomb pattern (Figure 1B). Immunohistochemical stains demonstrated diffuse positivity for CD34 antigen in the spindle cells (Figure 1C), but negativity for smooth muscle actin and Signal Transducer and Activator of Transcription 6 (STAT6). These findings confirmed the diagnosis of atrophic DFSP. Further evaluation with MRI showed a 9.3 cm x 6.5 cm cutaneous-subcutaneous lesion with enhancement on the anterior right-to-middle chest wall, extending 1.0 cm in depth and near the pectoralis major muscle (Figure 1D). No metastasis was detected by MRI and computed tomography (CT).

**Figure 2:**
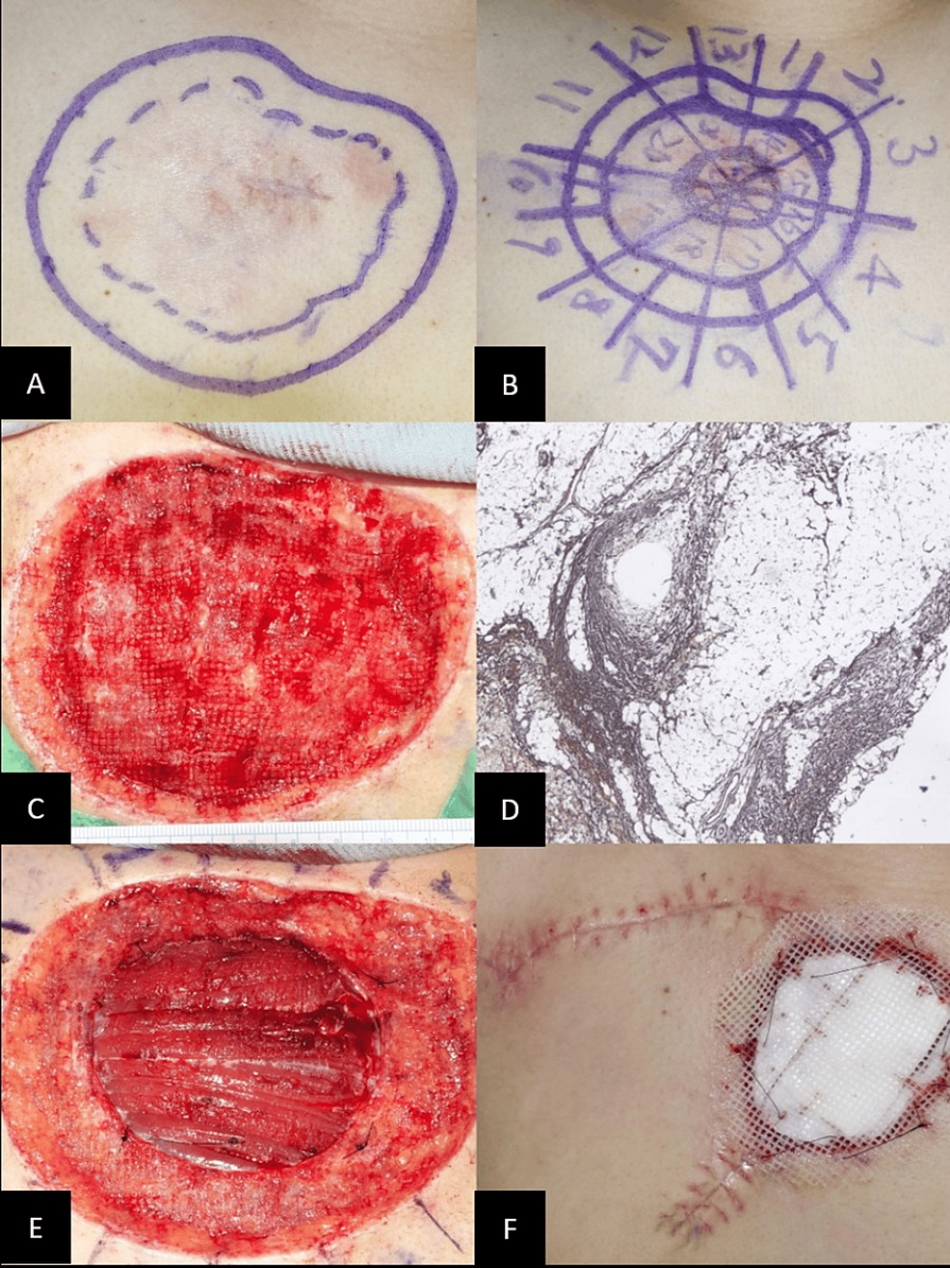
(A) 1.5 cm surgical safety margin; (B) stage I mapping of Mohs micrographic surgery; (C) skin defect after stage I Mohs surgery; (D) immunohistochemical stains demonstrated satellite positivity for CD34 antigen; (E) removal of deep fascia layer of pectoralis major muscle during stage II Mohs surgery; (F) the remaining defect is covered with artificial dermis.

The patient underwent slow MMS with a 1 cm surgical safety margin deep to the superficial fascia (Figures 2A-2C). Due to the involvement of the deep central margin, a stage II Mohs surgery was performed to ensure complete removal down to the superficial fascia of the pectoralis major muscle (Figures 2D-2E). Pathology confirmed no residual tumor. To reconstruct the resulting 13 cm x 12 cm defect, we employed a rotational flap combined with synthetic xenogeneic artificial dermis Terudermis (Olympus Terumo Biomaterials Corp., Tokyo, Japan) (Figure 2F).

## Discussion

Less than 5% of DFSP cases metastasize to regional lymph nodes or distant organs. However, the infiltration of surrounding tissue by the DFSP remains a major concern, particularly due to the high local recurrence rate associated with larger tumors [6]. Negative surgical margins are crucial for prognosis, with wide local excision (WLE) typically requiring at least a 3 cm lateral and a deep margin for complete resection [7]. MMS has been recently advocated over WLE for its superior ability to detect microscopic tumors and reduce recurrence rates in DFSP [8]. Slow Mohs is a modified form of MMS that uses paraffin-embedded tissue sections instead of frozen sections. This approach is preferred because extremely subtle tumor strands are more easily identifiable on paraffin-embedded sections than on frozen ones [9]. Slow Mohs was also advocated in the treatment of DFSP due to improved morphology, tissue conservation, and immunocytochemical labeling for tumor strands [10].

Skin grafts and local flaps are viable options for reconstruction following MMS. Common regional flap choices for chest wall reconstruction include the pectoralis major muscle flap or myocutaneous flaps. Over the past three decades, artificial dermis has proven successful in reconstructive surgery by facilitating wound bed preparation, enhancing healing quality, and reducing contraction and scar formation by extracellular matrix remodeling and elastin regeneration [11]. Additional benefits include immediate availability, extensive defect coverage, early recovery, and favorable cosmetic outcomes. In this case, we utilized Terudermis, a synthetic xenogeneic artificial dermis with a lyophilized collagen sponge reconstituted from heat-denatured bovine dermal type 1 collagen, as an acellular dermal scaffold that promotes cellular ingrowth [12]. It offers a reconstructive alternative to more extensive treatment utilizing flap transfer, which accompanies donor-site morbidity.

## Conclusions

We reported a case of atrophic DFSP with deep fascia involvement and highlighted the critical role of slow MMS in accurately identifying residual tumor strands. Furthermore, we emphasized the practical application and clinical benefits of employing artificial dermis for reconstructive purposes

## References

[1] Bowne WB, Antonescu CR, Leung DH (2000). Dermatofibrosarcoma protuberans: a clinicopathologic analysis of patients treated and followed at a single institution. Cancer.

[2] Acosta AE, Vélez CS (2017). Dermatofibrosarcoma protuberans. Curr Treat Options Oncol.

[3] Trinidad CM, Wangsiricharoen S, Prieto VG, Aung PP (2023). Rare variants of dermatofibrosarcoma protuberans: clinical, histologic, and molecular features and diagnostic pitfalls. Dermatopathology (Basel).

[4] Valdivielso-Ramos M, Torrelo A, Campos M, Feito M, Gamo R, Rodriguez-Peralto JL (2014). Pediatric dermatofibrosarcoma protuberans in Madrid, Spain: multi-institutional outcomes. Pediatr Dermatol.

[5] Rutkowski P, Debiec-Rychter M (2015). Current treatment options for dermatofibrosarcoma protuberans. Expert Rev Anticancer Ther.

[6] Baig IT, Lauck K, Nguyen QD (2023). Tumor size is the most significant risk factor for local recurrence in dermatofibrosarcoma protuberans: a large-scale retrospective cohort analysis. J Am Acad Dermatol.

[7] Kim BJ, Kim H, Jin US, Minn KW, Chang H (2015). Wide local excision for dermatofibrosarcoma protuberans: a single-center series of 90 patients. Biomed Res Int.

[8] Malan M, Xuejingzi W, Quan SJ (2019). The efficacy of Mohs micrographic surgery over the traditional wide local excision surgery in the cure of dermatofibrosarcoma protuberans. Pan Afr Med J.

[9] Hafner J, Schütz K, Morgenthaler W, Steiger E, Meyer V, Burg G (1999). Micrographic surgery ("slow Mohs") in cutaneous sarcomas. Dermatology.

[10] Orchard GE, Shams M (2012). Dermatofibrosarcoma protuberans: dealing with slow Mohs procedures employing formalin-fixed, paraffin wax-embedded tissue in a busy diagnostic laboratory. Br J Biomed Sci.

[11] Lamme EN, de Vries HJ, van Veen H, Gabbiani G, Westerhof W, Middelkoop E (1996). Extracellular matrix characterization during healing of full-thickness wounds treated with a collagen/elastin dermal substitute shows improved skin regeneration in pigs. J Histochem Cytochem.

[12] Soejima K, Chen X, Nozaki M, Hori K, Sakurai H, Takeuchi M (2006). Novel application method of artificial dermis: one-step grafting procedure of artificial dermis and skin, rat experimental study. Burns.

